# Effect of Thioridazine on Erythrocytes

**DOI:** 10.3390/toxins5101918

**Published:** 2013-10-23

**Authors:** Elisabeth Lang, Paola Modicano, Markus Arnold, Rosi Bissinger, Caterina Faggio, Majed Abed, Florian Lang

**Affiliations:** 1 Department of Physiology, Eberhard-Karls-University of Tuebingen, Gmelinstr.5, Tuebingen 72076, Germany; E-Mails: lilalu1@gmx.de (E.L.); paolamodicano@libero.it (P.M.); mail.markus.arnold@googlemail.com (M.A.); ro.bissinger@gmx.de (R.B.); dr.magd81@hotmail.com (M.A.); 2 Department of Biological and Environmental Sciences, University of Messina, Viale Ferdinando Stagno d’Alcontres, 31, S. Agata-Messina 98166, Italy; E-Mail: cfaggio@unime.it

**Keywords:** phosphatidylserine, thioridazine, calcium, cell volume, eryptosis

## Abstract

Background: Thioridazine, a neuroleptic phenothiazine with antimicrobial efficacy is known to trigger anemia. At least in theory, the anemia could result from stimulation of suicidal erythrocyte death or eryptosis, which is characterized by cell shrinkage and by phospholipid scrambling of the cell membrane with phosphatidylserine exposure at the erythrocyte surface. Triggers of eryptosis include increase of cytosolic Ca^2+^-concentration ([Ca^2+^]_i_) and activation of p38 kinase. The present study explored, whether thioridazine elicits eryptosis. Methods: [Ca^2+^]_i_ has been estimated from Fluo3-fluorescence, cell volume from forward scatter, phosphatidylserine exposure from annexin-V-binding, and hemolysis from hemoglobin release. Results: A 48 hours exposure to thioridazine was followed by a significant increase of [Ca^2+^]_i_ (30 µM), decrease of forward scatter (30 µM), and increase of annexin-V-binding (≥12 µM). Nominal absence of extracellular Ca^2+^ and p38 kinase inhibitor SB203580 (2 µM) significantly blunted but did not abolish annexin-V-binding following thioridazine exposure. Conclusions: Thioridazine stimulates eryptosis, an effect in part due to entry of extracellular Ca^2+^ and activation of p38 kinase.

## 1. Introduction

Thioridazine, a phenothiazine drug, has both antipsychotic efficacy [1,2] and anti-microbial activity [2]. It is particularly useful for the treatment of multidrug resistant tuberculosis [3,4,5,6].

Side effects of thioridazine include anemia [7,8]. Drug induced anemia could, at least in theory, result from stimulation of suicidal erythrocyte death or eryptosis [9]. Eryptotic erythrocytes are rapidly cleared from circulating blood and are, thus, removed prior to hemolysis [9]. The most important hallmark of eryptosis is breakdown of phosphatidylserine asymmetry of the erythrocyte cell membrane with translocation of phosphatidylserine to the erythrocyte surface [9]. Eryptosis is further typically paralleled by erythrocyte shrinkage [10]. Eryptosis may be triggered by increased cytosolic Ca^2+^ concentration ([Ca^2+^]_i_) due to Ca^2+^ entry through Ca^2+^-permeable cation channels [11,12], or due to permeabilization of the erythrocyte membrane [13]. Increased [Ca^2+^]_i_ leads to cell shrinkage due to activation of Ca^2+^-sensitive K^+^ channels [14], K^+^ exit, hyperpolarization, Cl^−^ exit and thus cellular loss of KCl and osmotically obliged water [10]. Increased [Ca^2+^]_i_ further leads to translocation of phosphatidylserine from the inner leaflet of the cell membrane to the erythrocyte surface [15]. The sensitivity of cell membrane scrambling to cytosolic Ca^2+^ is enhanced by ceramide [16]. Eryptosis is further triggered by energy depletion [17] and caspase activation [18,19,20,21,22]. The cytosolic machinery governing eryptosis further involves AMP activated kinase AMPK [12], cGMP-dependent protein kinase [23], Janus-activated kinase JAK3 [24], casein kinase [25,26], p38 kinase [27], PAK2 kinase [28], as well as sorafenib [29] and sunifinib [30] sensitive kinases.

Eryptosis may be triggered by a wide variety of xenobiotics [30,31,32,33,34,35,36,37,38,39,40,41,42,43,44,45,46,47,48,49,50,51,52,53,54,55,56,57,58,59,60,61]. Moreover, excessive eryptosis contributes to the pathophysiology of several clinical disorders [9], such as diabetes [22,62,63], renal insufficiency [64], hemolytic uremic syndrome [65], sepsis [66], malaria [67,68,69,70,71], sickle cell disease [72], Wilson’s disease [70], iron deficiency [73], malignancy [74], phosphate depletion [75], and metabolic syndrome [57].

The present study explored, whether thioridazine triggers eryptosis. To this end, the effect of thioridazine on [Ca^2+^]_i_, cell volume and phosphatidylserine abundance at the erythrocyte surface has been determined.

## 2. Results and Discussion

**Figure 1:**
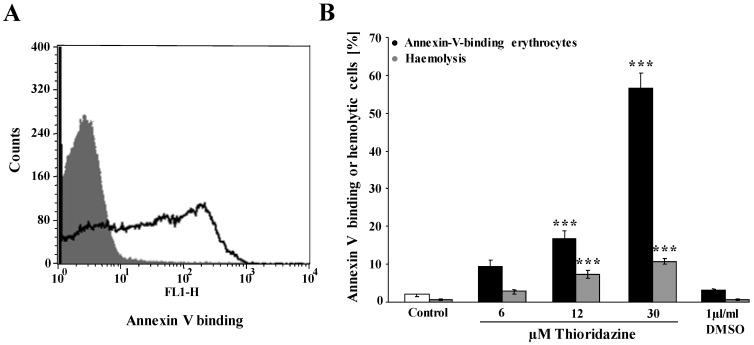
Effect of thioridazine on phosphatidylserine exposure and hemolysis.(**A**) Original histogram of annexin V binding of erythrocytes following exposure for 48 h to Ringer solution without (grey shadow) and with (black line) presence of 30 µM thioridazine; (**B**) Arithmetic means ± SEM (*n* = 6) of erythrocyte annexin-V-binding following incubation for 48 h to Ringer solution without (white bar) or with (black bars) presence of thioridazine (6–30 µM). For comparison, arithmetic means ± SEM (*n* = 5) of the percentage of hemolysis is shown as grey bars. *** (*p* < 0.001) indicate significant differences from the absence of thioridazine (ANOVA).

The present study explored whether thioridazine triggers eryptosis, the suicidal death of erythrocytes. The key hallmark of eryptosis is the triggering of cell membrane scrambling with increase of phosphatidylserine abundance at the cell surface. Accordingly, phosphatidylserine exposing erythrocytes were identified by annexin-V-binding in flow cytometry. As shown in Figure 1, treatment of human erythrocytes from healthy individuals with thioridazine increased the percentage of annexin-V-binding erythrocytes, an effect reaching statistical significance at 12 µM thioridazine concentration (Figure 1A,B). In order to explore whether thioridazine treatment may trigger hemolysis, the percentage of hemolysed erythrocytes was estimated from the hemoglobin concentration in the supernatant. As illustrated in Figure 1B, thioridazine treatment significantly increased the hemoglobin concentration in the supernatant. The percentage of hemolytic erythrocytes was, however, clearly smaller than the percentage of annexin V binding erythrocytes (Figure 1B).

**Figure 2:**
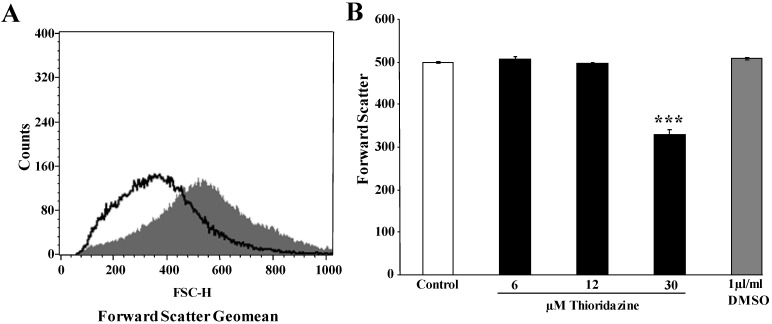
Effect of thioridazine on erythrocyte forward scatter. (**A**) Original histogram of forward scatter of erythrocytes following exposure for 48 h to Ringer solution without (grey shadow) and with (black line) presence of 30 µM thioridazine; (**B**) Arithmetic means ± SEM (*n* = 6) of the normalized erythrocyte forward scatter (FSC) following incubation for 48 h to Ringer solution without (white bar) or with (black bars) thioridazine (6–30 µM); *** (*p* < 0.001) indicates significant difference from the absence of thioridazine (ANOVA).

Another hallmark of eryptosis is cell shrinkage. Thus, cell volume was estimated utilizing forward scatter in flow cytometry. As illustrated in Figure 2A,B, thioridazine treatment resulted in a decrease of forward scatter, an effect reaching statistical significance at a 30 µM thioridazine concentration.

**Figure 3:**
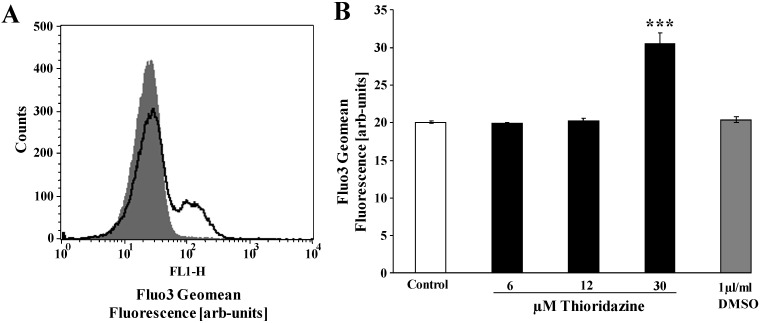
Effect of thioridazine on erythrocyte cytosolic Ca^2+^ concentration. (**A**) Original histogram of Fluo3 fluorescence in erythrocytes following exposure for 48 h to Ringer solution without (grey shadow) and with (black line) presence of 30 µM thioridazine; (**B**) Arithmetic means ± SEM (*n* = 6) of the Fluo3 fluorescence (arbitrary units) in erythrocytes exposed for 48 h to Ringer solution without (white bar) or with (black bars) thioridazine (6–30 µM); *** (*p* < 0.001) indicates significant difference from the absence of thioridazine (ANOVA).

Both, cell membrane scrambling and cell shrinkage could have resulted from increase of cytosolic Ca^2+^ concentration ([Ca^2+^]_i_). Accordingly, [Ca^2+^]_i_ was determined utilizing Fluo3 fluorescence. To this end, erythrocytes were loaded with Fluo3-AM and Fluo3 fluorescence determined in flow cytometry following prior incubation of the erythrocytes in Ringer solution without or with thioridazine. As illustrated in Figure 3A,B, treatment of human erythrocytes with thioridazine increased Fluo3 fluorescence, an effect reaching statistical significance at a 30 µM thioridazine concentration.

**Figure 4:**
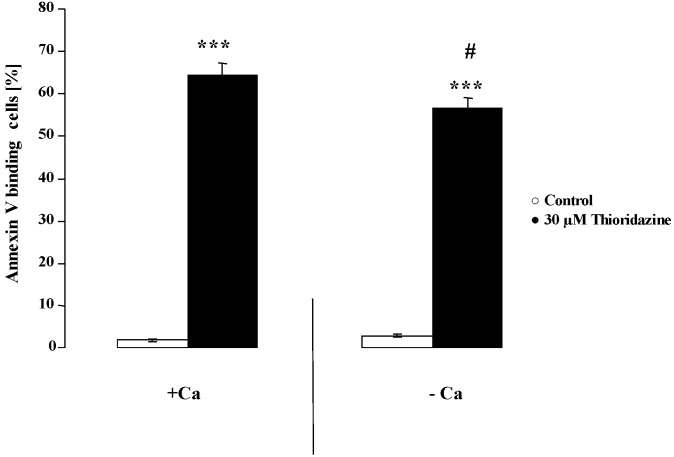
Effect of Ca^2+^ withdrawal on thioridazine induced annexin-V-binding.Arithmetic means ± SEM (n = 7) of the percentage of annexin-V-binding erythrocytes after a 48 h treatment with Ringer solution without (white bars) or with (black bars) 30 µM thioridazine in the presence (left bars, +Ca) and absence (right bars, −Ca) of calcium. *** (*p* < 0.001) indicates significant difference from the absence of thioridazine (ANOVA); # (*p* < 0.05) indicates significant difference from the respective values in the presence of Ca^2+^ (ANOVA).

In order to explore whether extracellular Ca^2+^ entry was required for the effect of thioridazine on cell membrane scrambling, erythrocytes were exposed to 30 µM of thioridazine for 48 hours, either in the presence of 1 mM Ca^2+^ or in the absence of Ca^2+^ and presence of Ca^2+^ chelator EGTA (1 mM). As shown in Figure 4, the effect of thioridazine on annexin-V-binding was significantly blunted in the nominal absence of Ca^2+^. However, even in the absence of extracellular Ca^2+^, thioridazine still significantly increased the percentage of annexin-V-bindiung erythrocytes pointing to additional mechanisms involved.

**Figure 5:**
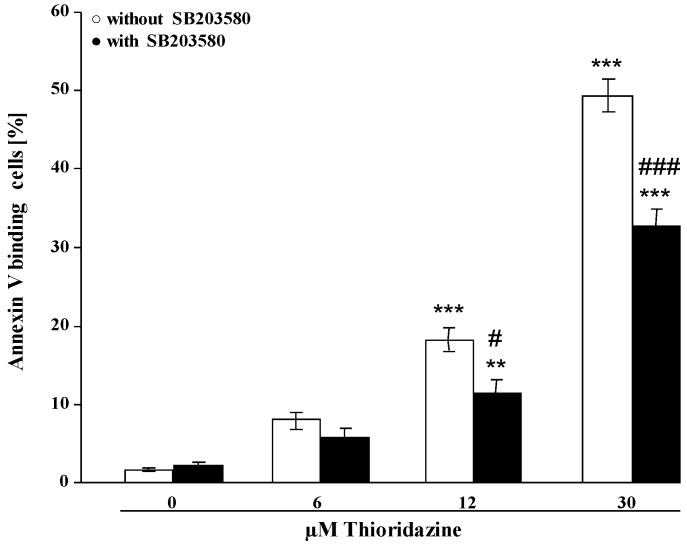
Effect of thioridazine on phosphatidylserine exposure in the presence or absence of p38 kinase inhibitor SB203580. Arithmetic means ± SEM (*n* = 6) of erythrocyte annexin-V-binding following incubation for 48 h to Ringer solution without or with presence of thioridazine (6–30 µM) in the absence (white bars) or presence (black bars) of 2 µM SB203580. ** (*p* < 0.01); *** (*p* < 0.001) indicate significant differences from the absence of thioridazine (ANOVA); # (*p* < 0.05); ### (*p* < 0.001) indicate significant differences from the absence of SB203580 (ANOVA).

In order to explore whether the additional mechanisms could include p38 kinase, erythrocytes were exposed in further experiments to 6–30 µM of thioridazine for 48 h in either the presence or absence of the p38 kinase inhibitor SB203580 (2 µM). As shown in Figure 5, the effect of thioridazine on annexin-V-binding was significantly blunted in the presence of SB203580. However, even in the presence of SB203580, thioridazine still significantly increased the percentage of annexin-V-binding erythrocytes.

**Figure 6:**
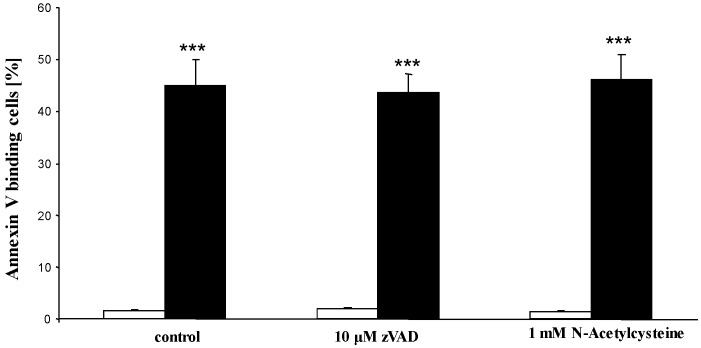
Effect of thioridazine on phosphatidylserine exposure in the presence or absence of pancapsase inhibitor zVAD or antioxidant *N*-acetylcysteine. Arithmetic means ± SEM (*n* = 4 each) of erythrocyte annexin-V-binding following incubation for 48 h to Ringer solution without (white bars) or with (black bars) presence of thioridazine (30 µM) in the absence (control, left bars) or presence of 10 µM zVAD (middle bars) or 1 mM *N*-acetylcysteine (right bars). ** (*p* < 0.01); *** (*p* < 0.001) indicate significant differences from the absence of thioridazine (ANOVA); # (*p* < 0.05); ### (*p* < 0.001) indicate significant differences from the absence of SB203580 (ANOVA).

Additional experiments were performed in the absence and presence of pancaspase inhibitor zVAD (10 µM). As illustrated in Figure 6, a 48 h exposure to thioridazine (30 µM) increased the percentage of annexin V binding erythrocytes to a similar value in the presence and absence of zVAD. Further experiments were performed in the absence and presence of antioxidant *N*-acetyl-cysteine (1 mM). As a result, a 48 h exposure to thioridazine (30 µM) increased the percentage of annexin V binding erythrocytes again to a similar value in the presence and absence of N-acetyl-cysteine.

The present study demonstrates a novel effect of thioridazine, *i.e.*, the stimulation of eryptosis, the suicidal erythrocyte death. Exposure of human erythrocytes to thioridazine decreased cell volume and triggered erythrocyte of eryptosis. The concentrations required to trigger eryptosis were within the range of concentrations (6 µg/mL ≈ 15 µM) encountered *in vivo* [76].

Thioridazine influenced erythrocyte cell volume most likely by stimulating entry and/or impairing extrusion of Ca^2+^ with subsequent increase of cytosolic Ca^2+^ concentration ([Ca^2+^]_i_), activation of Ca^2+^ sensitive K^+^ channels [14,77], K^+^ exit, cell membrane hyperpolarisation, Cl^−^ exit and cellular loss of KCl with osmotically obliged water [10]. The Ca^2+^ entry may have been accomplished by activation of the endogenous Ca^2+^ permeable non-selective cation channels [11], which are known to be activated by oxidative stress [78]. Ca^2+^ is extruded by the Ca^2+^ ATPase, which has previously been shown to be inhibited by thioridazine [79]. Stimulation of extracellular Ca^2+^ entry with subsequent increase of [Ca^2+^]_i_ further contributed to the triggering of cell membrane scrambling by thioridazine. However, even in the absence of extracellular Ca^2+^ thioridazine still significantly triggered cell membrane scrambling, an observation pointing to the involvement of additional mechanisms. Mechanisms mediating the stimulation of eryptosis following osmotic shock include p38 kinase [27], which is expressed in human erythrocytes and is activated by hyperosmotic shock, a known trigger of eryptosis [27]. As shown earlier [27], p38 kinase is phosphorylated upon osmotic shock and inhibitors of the kinase significantly blunt the decrease of forward scatter and the increase of annexin-V-binding following osmotic shock. Inhibition of p38 kinase significantly blunted but did not abrogate thioridazine induced cell membrane scrambling again indicating that p38 kinase contributes to but does not fully account for the stimulation of eryptosis. Thus, the present results do not rule out the involvement of further cellular mechanisms paticipating in the triggering of eryptosis by thioridazine.

Whatever mechanisms involved in the triggering of phosphatidylserine translocation by thioridazine, phosphatidylserine exposed at the surface of eryptotic cells fosters the binding of the affected erythrocytes to phagocytosing cells with subsequent engulfment and thus clearance of those cells [16]. The clearance of eryptotic erythrocytes from circulating blood may lead to anemia [9].

Phosphatidylserine exposing erythrocytes may further adhere to endothelial CXCL16/SR-PSO of the vascular wall [80] and, thus, interfere with blood flow [80,81,82,83,84,85]. Phosphatidylserine exposing erythrocytes could in addition foster blood clotting and, thus, cause thrombosis [81,86,87].

Besides causing anemia [7,8], thioridazine intoxication leads to impairment of consciousness, cardiac arrhythmia and subsequent cardiac failure with pulmonary edema, severe hypotension, and renal failure [88,89,90]. At least in theory, similar mechanisms may be effective in the derangement of cardiac excitation and suicidal erythrocyte death. Notably, pathogenesis of cardiac arrhythmia may involve activation of p38 kinase [91,92]. However, whether or not thioridazine activates p38 kinase in nucleated cells, remains to be shown.

On the other hand, triggering of eryptosis may result in the clearance of defective erythrocytes prior to rupture of the cell membrane and thus prevent release of cellular hemoglobin, which is filtered in renal glomerula and subsequently occludes renal tubules [93].

## 3. Experimental Section

### 3.1. Erythrocytes, Solutions and Chemicals

Leukocyte-depleted erythrocytes were kindly provided by the blood bank of the University of Tuebingen. The study is approved by the ethics committee of the University of Tuebingen (184/2003V). Erythrocytes were incubated *in vitro* at a hematocrit of 0.4% in Ringer solution containing (in mM) 125 NaCl, 5 KCl, 1 MgSO4, 32 *N*-2-hydroxyethylpiperazine-*N*-2-ethanesulfonic acid (HEPES), 5 glucose, 1 CaCl_2_; pH 7.4 at 37 °C for 48 h. Where indicated, erythrocytes were exposed to thioridazine (Sigma, Aldrich, Germany) at the indicated concentrations (dissolved in 0.001% DMSO). In Ca^2+^-free Ringer solution, 1 mM CaCl_2_ was substituted by 1 mM glycol-bis(2-aminoethylether)-*N*,*N*,*N*',*N*'-tetraacetic acid (EGTA) and/or p38 kinase inhibited by addition of SB203580 (2 μM; Tocris, Bristol, UK).

### 3.2. FACS Analysis of Annexin-V-Binding and Forward Scatter

After incubation under the respective experimental condition, 50 µL cell suspension were washed in Ringer solution containing 5 mM CaCl_2_ and then stained with Annexin-V-FITC (1:200 dilution; ImmunoTools, Friesoythe, Germany) in this solution at 37 °C for 20 min under protection from light. In the following, the forward scatter (FSC) of the cells was determined, and annexin-V fluorescence intensity was measured with an excitation wavelength of 488 nm and an emission wavelength of 530 nm utilizing a FACS Calibur (BD, Heidelberg, Germany).

### 3.3. Measurement of Intracellular Ca^2+^

After incubation, erythrocytes were washed in Ringer solution and then loaded with Fluo-3/AM (Biotium, Hayward, CA, USA) in Ringer solution containing 5 mM CaCl_2_ and 5 µM Fluo-3/AM. The cells were incubated at 37 °C for 30 min and washed twice in Ringer solution containing 5 mM CaCl_2_. The Fluo-3/AM-loaded erythrocytes were resuspended in 150 µL Ringer. Following this, Ca^2+^-dependent fluorescence intensity was measured with an excitation wavelength of 488 nm and an emission wavelength of 530 nm utilizing a FACS Calibur (BD, Heidelberg, Germany).

### 3.4. Measurement of Hemolysis

For the determination of hemolysis the samples were centrifuged (3 min at 400 g, room temperature) after incubation, and the supernatants were harvested. As a measure of hemolysis, the hemoglobin (Hb) concentration of the supernatant was determined photometrically at 405 nm. The absorption of the supernatant of erythrocytes lysed in distilled water was defined as 100% hemolysis.

### 3.5. Statistics

Data are expressed as arithmetic means ± SEM. As indicated in the Figure legends, statistical analysis was made using ANOVA with Tukey’s test as post-test and t test as appropriate. n denotes the number of different erythrocyte specimens studied. Since different erythrocyte specimens used in distinct experiments are differently susceptible to triggers of eryptosis, the same erythrocyte specimens have been used for control and experimental conditions.

## 4. Conclusions

In conclusion, thioridazine is shown to stimulate eryptosis, which is characterized by cell membrane scrambling and cell shrinkage. The substance is partially effective by activation of p38 kinase and by increase of cytosolic Ca^2+^ concentration.
